# Lower dose of ATG combined with basiliximab for haploidentical hematopoietic stem cell transplantation is associated with effective control of GVHD and less CMV viremia

**DOI:** 10.3389/fimmu.2022.1017850

**Published:** 2022-11-15

**Authors:** Zhenli Huang, Han Yan, Yao Teng, Wei Shi, Linghui Xia

**Affiliations:** Institute of Hematology, Union Hospital, Tongji Medical College, Huazhong University of Science and Technology, Wuhan, China

**Keywords:** ATG, basiliximab, GVHD prophylaxis, haploidentical HSCT, hematologic malignancies

## Abstract

Currently, the graft-versus-host disease (GVHD) prophylaxis consists of an immunosuppressive therapy mainly based on antithymocyte globulin (ATG) or post-transplant cyclophosphamide (PTCy). GVHD remains a major complication and limitation to successful allogeneic haploidentical hematopoietic stem cell transplantation (haplo‐HSCT). We modified the ATG-based GVHD prophylaxis with the addition of basiliximab in the setting of haplo-HSCT and attempted to explore the appropriate dosages. We conducted a retrospective analysis of 239 patients with intermediate- or high-risk hematologic malignancies who received haplo-HSCT with unmanipulated peripheral blood stem cells combined or not with bone marrow. All patients received the same GVHD prophylaxis consisting of the combination of methotrexate, cyclosporine or tacrolimus, mycofenolate-mofetil, and basiliximab with different doses of ATG (5-9mg/kg). With a median time of 11 days (range, 7-40 days), the rate of neutrophil engraftment was 96.65%. The 100-day cumulative incidences (CIs) of grade II–IV and III–IV aGVHD were 15.8 ± 2.5% and 5.0 ± 1.5%, while the 2-year CIs of total cGVHD and extensive cGVHD were 9.8 ± 2.2% and 4.1 ± 1.5%, respectively. The 3-year CIs of treatment-related mortality (TRM), relapse, overall survival (OS), and disease-free survival (DFS) were 14.6 ± 2.6%, 28.1 ± 3.4%, 60.9 ± 3.4%, 57.3 ± 3.4%, respectively. Furthermore, the impact of the reduction of the ATG dose to 6 mg/kg or less in combination with basiliximab on GVHD prevention and transplant outcomes among patients was analyzed. Compared to higher dose of ATG(>6mg/kg), lower dose of ATG (≤6mg/kg) was associated with a significant reduced risk of CMV viremia (52.38% *vs* 79.35%, *P*<0.001), while the incidences of aGVHD and cGVHD were similar between the two dose levels. No significant effect was found with regard to the risk of relapse, TRM, and OS. ATG combined with basiliximab could prevent GVHD efficiently and safely. The optimal scheme of using this combined regimen of ATG and basiliximab is that administration of lower dose ATG (≤6mg/kg), which seems to be more appropriate for balancing infection control and GVHD prophylaxis.

## Introduction

Haploidentical hematopoietic stem cell transplant (haplo-HSCT) has been applied with promising results for patients diagnosed with hematological malignancies lacking an appropriately matched sibling or unrelated donor and urgently requiring transplantation (1–5). Although considerable progress has been made to overcome the challenging human leukocyte antigen (HLA)-barriers in the setting of haplo-HSCT through various graft-versus-host disease (GVHD) prevention approaches, GVHD remains a major factor contributing to variable degrees of transplantation-related morbidity and mortality, as well as quality of life compromise. Currently, T-replete strategies using unmanipulated allografts have been the dominant procedures for haplo-HSCT, in which the GVHD prophylaxis consists of an immunosuppressive therapy mainly based on antithymocyte globulin (ATG) or post-transplant cyclophosphamide (PTCy) (5–12). Both ATG and PTCy have consistently demonstrated efficacies in GVHD prophylaxis, but each strategy uniquely affects post-transplant immune reconstitution resulting in concordant increased incidences of opportunistic infections and malignancy relapse (9, 11, 13–15). The optimal approach to manipulate the delicate balance between controlling GVHD and timely T-cell immune reconstitution remains uncertain and has become a main focus of attention.

Basiliximab is a chimeric murine-human monoclonal antibody that is directed against the IL-2Rα chain (CD25) to inhibit T lymphocyte activation. Compared with ATG, basiliximab has similar immunosuppressive efficacies but leads to a lower incidence of infection and infection-related mortality in renal transplantation and heart transplantation (16–18). In terms of HSCT, basiliximab has been used to treat acute steroid-refractory GVHD with satisfactory responses (19–23). Considering that basiliximab only selectively eliminates the donor-specific alloreactive T cells without affecting the resting T cells present in the graft (24), basiliximab may prevent GVHD without compromising immune function. In fact, the protective effect of CD25 blockade on GVHD is controversial. It has been reported that prophylactic administration of daclizumab (another CD25 blockade) does not prevent acute GVHD (aGVHD) but may increase the risk of chronic GVHD (cGVHD) (25). On the contrary, we and others have indicated that basiliximab alone or with the combination of ATG contributes to GVHD prophylaxis in HSCT without increasing infections (26–32). Nevertheless, these studies contained a relatively small number of patients; and a larger study with more patients and longer follow-up is warranted. Therefore, we modified the ATG-based GVHD prophylaxis with the addition of basiliximab in the setting of haplo-HSCT. Furthermore, in order to reduce the risk of GVHD without increasing the incidence of infection events or compromising overall survival, and simultaneously decrease expense related to ATG, we attempted to explore the appropriate dose of ATG combined with basiliximab.

In this study, we retrospectively analyzed a large series of patients with hematological malignancies who underwent haplo-HSCT using GVHD immunosuppressive prophylaxis with different dosages of ATG incorporated with basiliximab. On the one hand, we investigated the efficacy and safety of this combined GVHD prophylaxis option. On the other hand, we performed a preliminary assessment of the appropriate lower dose of ATG coupled with basiliximab in our haplo-HSCT system.

## Methods

### Patients

A cohort of 239 consecutive patients were included in this retrospective study. These patients received their haplo-HSCT from February 2013 to January 2019 at the Institute of Hematology, Union Hospital, Tongji Medical College, Huazhong University of Science and Technology, China. The study was reviewed and approved by the Ethical Committee of Huazhong University of Science and Technology, and informed consents were provided by all patients or legal guardians. The eligibility criteria included patients diagnosed with intermediate- or high-risk hematologic malignancies who received haplo-HSCT. Patients with HLA matching rates of 10/10, 9/10 and 6/6 were excluded. Patients were stratified at intermediate or high risk as we described previously (28, 33, 34). High-risk ALL was defined as beyond first complete remission (CR1) and at least one of the following criteria at diagnosis: age >35 years; high white blood cell count (>30 × 10^9^/L for B-lineage ALL, >100 × 10^9^/L for T-lineage ALL); poor-risk cytogenetics (ph+, t(4;11), t(8;14), complex karyotype, or low hypodiploidy near triploidy); delayed CR1 (>28 days of induction therapy). The definition of high-risk AML was conformed to the following criteria at diagnosis: hyperleukocytosis at diagnosis; no response to induction chemotherapy; relapse within 6 months after induction or consolidation therapy; ≥2 relapses or relapse after auto-HSCT; secondary AML; ≥CR2 or in no remission; poor cytogenetics according to the National Comprehensive Cancer Network 2018 guidelines (www.nccn.org).

### HLA matching and stem cell source

As previously defined (28), HLA class I and class II were detected in all donor/recipient pairs. For donor/recipient pairs in 6 loci (HLA-A, -B, -DRB1), HLA-A and HLA-B were typed by intermediate-resolution DNA techniques, and HLA-DRB1 was performed through high resolution DNA techniques. For donor/recipient pairs in 10 loci (HLA-A, -B, -C, -DRB1, -DQB1), HLA typing was performed in high-resolution DNA techniques. Donors were ranked according to HLA matching sites, young age, male, negative donor-specific antibodies (DSA), good physical condition, and blood type matching. Bone marrow (BM)stem cells and/or peripheral blood stem cells (PBSCs) were collected from donors according to standard mobilization protocols. Donors were administrated rhG-CSF(8-10μg/kg/day) by continuous subcutaneous injections for six days (from day -3 to day 0 during transplantation). For HLA 4-5/6 or 6-7/10 matched related donor transplantation, G-CSF-mobilized PBSCs were infused into the recipient after collection on the fourth to sixth day after subcutaneous administration. For the donor-recipient HLA 3/6 or 5/10-matched setting, G-CSF mobilized BM was harvested on the fourth day and G-CSF primed PBSCs were collected on the fifth and the sixth day after subcutaneous administration. If the target count for CD34^+^cells was not above 4×10^6^/kg of the recipient weight, the mobilization period would be extended by one day. BM stem cells were extracted from the donor under general anesthesia in the operation room.

### Conditioning regimen and GVHD prophylaxis

Conditioning regimens included in this study were divided into two categories: intensified myeloablative conditioning regimens (IMC) and myeloablative conditioning regimens (MAC). The details were described in our previous studies (28, 33, 34). All patients were administrated with a combination of cyclosporine (CsA) or tacrolimus, short-term methotrexate (MTX), mycofenolate-mofetil (MMF), ATG, and basiliximab for GVHD prophylaxis. The GVHD prophylaxis was described as follows: CsA 5mg/kg or tacrolimus 0.5mg/kg (twice daily) was given from day -1 until day +180. Individualized dosage adjustment of CsA or tacrolimus was based on plasma concentration to maintain a target dose (CsA:150–250 ng/mL, tacrolimous:10-15ng/ml). MTX was administered intravenously at dosages of 15 mg/m2 on day +1 and 10 mg/m2 on days +3, +6 and +11. MMF (7.5 mg/kg, orally twice daily) was administered from day +7, which was tapered to half until day +60 and was discontinued based on the presence or absence of severe GVHD, infectious diseases, and relapse risk. Basiliximab was given intravenously at a dose of 20 mg by 30-minute IV infusion on day 0 (2 hours before graft infusion) and day +4. ATG (rabbit anti-human thymocyte immunoglobulin, Sanofi Aventis, Paris, France) was given with a median total dose of 6mg/kg (range, 5 to 9) from day -3 until day -1. For donor-recipient HLA 4-5/6matched or HLA 6-7/10matched setting, patients received ATG at a total dose of 6 mg/kg. For the HLA 3/6 or 5/10 matched transplant, a total dose of 9 mg/kg ATG was used (28). If the patient was unable to tolerate a predetermined dose of ATG during the transplant procedure, the dose of ATG was reduced. Diagnosis and clinical grading of aGVHD and cGVHD were established according to the standard criteria (35, 36). GVHD prophylaxis was summarized in **Supplementary Figure 1**.

### Supportive care

All patients were hospitalized in laminar airflow rooms and given standard antimicrobial prophylaxis covering fungal and bacteria agents in our institution. Cytomegalovirus (CMV) DNA copies in plasma specimen were monitored weekly from day -7 until day +100, once every 2 weeks until day+180, and once per month until one year after HSCT. CMV DNA copies were monitored by polymerase chain reaction for all patients. The threshold for CMV-DNA copies were no more than 500 copies/mL in plasma specimen (33, 37). Pre-emptive ganciclovir or foscarnet therapy was initiated with the evidence of two consecutive positive tests without a sign of viral diseases and was continued until the CMV DNA monitoring was negative on two occasions.

### Definitions and endpoints

Overall survival (OS) was defined as the period from transplantation to the date of last follow-up or death due to any cause. Disease-free survival (DFS) was defined as the time from transplantation to either last follow-up or disease recurrence or death due to any causes. Data for patients who were alive or lost to follow-up were censored at the time of last contact. The time of neutrophil implantation was defined as from day 0 to the day when neutrophil count exceeded 0.5×10^9^/L for 3 consecutive days. The time of platelet implantation was defined as from day 0 to the day when platelet count exceeded 20×10^9^/L for 7 consecutive days without platelet transfusion. Treatment-related mortality (TRM) was defined as the time from transplantation to death due to any causes except disease recurrence/progression, considering relapse as the competing risk. The incidences of aGVHD and cGVHD were evaluated in all patients as described in the literature (30). GVHD/relapse-free survival (GRFS) was defined as the time from transplantation to last follow-up without grade III-IV aGVHD and/or cGVHD requiring immunosuppressive treatment and without relapse (38).

### Statistical analysis

Continuous variables were expressed as median with range, whereas categorical variables were presented as frequency and percentages. Statistical comparisons were made with the χ2 or Fisher exact test for categorical variables. If assumption of normality was not met for continuous variables, the Mann–Whitney U test was used as nonparametric test. Relapse, OS, DFS, TRM, and GRFS were estimated using the Kaplan–Meier methods and were compared by the log-rank tests. The Cox regression model was used for analyzing prognostic factors for aGVHD, cGVHD, relapse, OS, DFS, and TRM. TRM and relapse were the competing risks. For aGVHD and cGVHD, the competing events were relapse and death. A multivariate analysis was performed using Cox proportional hazards model. A two-tailed p value < 0.05 was considered to be statistically significant. Statistical analyses were performed with SPSS 25.0 and R version 4.1.2.

## Results

### Patient characteristics

Two hundred and thirty-nine patients with hematologic malignancies were included from February 2013 to January 2019. The overall characteristics of the patients and donors were summarized in **Table 1**. The study was divided into two groups according to the doses of ATG, including lower-dose group (ATG ≤ 6mg/kg) and higher-dose group (ATG >6mg/kg). Detailed ATG doses for the two groups were shown in the **Supplementary Table 1**. There were no significant differences between the groups in terms of patients’ age, gender, diagnosis, disease status at HSCT, risk stratification, donor/recipient gender match, ABO match, conditioning regimen, and median number of CD34^+^ cells in two groups. The median doses of infused nucleated and CD34^+^cells for the whole study were 15.40×10^8^/kg (7.08-48.35) and 6.06×10^6^/kg (1.65-25.1), respectively. The median follow-up for survivors in the lower-dose ATG group and higher-dose ATG group was 21.8 months (range, 9.6-74.1) and 36.3 months (range, 20.9-79.2), respectively.

**Table 1:** Patient and donor characteristics.

Baseline variable	All patients	ATG ≤ 6mg/kg	ATG>6mg/kg	*P* value
	(n=239)	(n=147)	(n=92)	
**Median age, years, (range)**				
Patient Donor	28(6-57) 36.5(7-60)	29(6-57) 39(7-59)	28(8-55) 34(9-60)	0.382 0.345
**Patient age, n (%)**				0.813
<18 y 18-49y ≥50y	37(15.48) 181(75.73) 21(8.79)	24(16.33) 108(73.47) 15(9.52)	13(14.13) 71(77.17) 8(8.70)	
**Patient gender, n (%)**				0.357
Male Female	149(62.34) 90(37.66)	95(64.63) 52(35.37)	54(58.70) 39(42.29)	
**Diagnosis (n, %)**	95(39.75)	56(38.10)	38(41.30)	0.534
ALL AML MDS	117(48.95) 27(11.30)	76(51.70) 15(10.20)	41(44.57) 12(13.04)	
**Disease status at HSCT (n, %)**				0.277
NR CR, MRD- CR, MRD+	27(11.30) 194(81.17) 18(7.53)	16(10.88) 123(64.63) 8(83.67)	11(11.96) 71(77.17) 10(10.87)	
**Risk stratification (n, %)**				0.159
High Intermediate	185(77.41) 54(22.59)	110(74.83) 37(25.17)	76(82.61) 16(17.39)	
**Donor (n, %)**				0.651
Parent Child Sibling	94(39.33) 52(21.76) 93(38.91)	59(40.14) 34(23.13) 54(36.73)	35(38.04) 18(19.57) 39(42.39)	
**HLA**				<0.001
**HLA(A/B/DR) (n, %)**				
3/6M 4-5/6M	93(38.91) 88(36.82)	10(6.80) 80(54.42)	83(90.21) 8(8.70)	
**HLA(A/B/C/DR/DQ) (n, %)**				
5/10M 6-7/10M	44(18.41) 14(5.85)	44(29.93) 13(8.84)	0(0.00) 1(1.09)	
**Stem cell source (n, %)**				<0.001
Peripheral blood stem cells Peripheral blood stem cells + bone marrow	126(52.72) 113(47.28)	103(70.07) 44(29.93)	23(25.00) 69(75.00)	
**Donor/recipient gender match**				0.109
Female to male	49(20.50)	35(23.81)	14(15.22)	
Others	190(79.50)	112(76.19)	78((84.78)	
**ABO match (n, %)**				0.267
Match Mismatch	101(42.26) 138(57.74)	58(39.46) 89(60.54)	43(46.74) 49(53.26)	
**Donor/recipient CMV serostatus (n, %)**				0.019
Negative/negative	230(96.23)	145(98.64)	85(92.39)	
Positive/negative	7(2.93)	1(0.68)	6(6.52)	
Negative/positive	2(0.84)	1(0.68)	1(1.09)	
Positive/positive	0(0.00)	0(0.00)	0(0.00)	
**Conditioning regimen (n, %)**				0.095
Intensified myeloablative	178(74.48)	104(70.75)	74(80.43)	
Myeloablative	61(25.52)	43(29.25)	18(19.57)	
**Median nucleated cells, ×10^8^/kg (range)** **Median CD34^+^ cells, × 10^6^/kg (range)**	15.40(7.08-48.35) 6.06(1.65-25.1)	14.63(7.08-48.35) 6.18(1.65-25.1)	16.88(7.26-35.21) 5.89(1.66-21.51)	0.021 0.452
**Median follow-up for survivors, months (range)**	28.9(9.6-79.2)	21.8(9.6-74.1)	36.3(20.9-79.2)	–

ALL acute lymphoblastic leukemia, AML acute myeloid leukemia, MDS myelodysplastic syndrome, HSCT hematopoietic stem cell transplantation, CR complete remission, NR no remission, MRD+ minimal residual disease positive; MRD- minimal residual disease negative, HLA human leukocyte antigen, CMV cytomegalovirus.

### Engraftment

Eight patients died without neutrophil and platelet recovery. Two hundred and thirty-one (96.65%) patients achieved neutrophil engraftment with a median time of 11 days (range, 7 to 40). Two hundred and twenty-seven (94.98%) patients achieved platelet engraftment with a median time of 12 days (range, 6 to 153). The median time to achieve neutrophil and platelet engraftment showed no statistical difference in different ATG doses groups (neutrophil: 11 days (8-21) *vs* 11 days (7-40), *P*=0.809; platelet: 12 days (7-100) *vs* 13 days (6-153), *P*=0.635, **Table 2**). Multivariate analysis showed that no remission at transplantation was the only independent risk factor for platelet engraftment (HR: 6.374, 95% confidence interval (CI): 1.821–22.315, *P*=0.004).

**Table 2:** Post-transplant information and outcomes.

Post-transplant information and outcomes	Overall (N=239)	ATG ≤ 6mg/kg (N=147)	ATG>6mg/kg (N=92)	*P* value
Engraftment: median days (range)				
Neutrophil	11 (7-40)	11(8-21)	11(7-40)	0.809
Platelet	12 (6-153)	12(7-100)	13(6-153)	0.365
Graft failure, n (%)	2(0.84)	0(0.00)	2(2.17)	–
Infectious complications, n (%)				
Pneumonia, n (%)	46(19.25)	34(23.13)	12(13.04)	0.054
CMV viremia, n (%)	150(62.76)	77(52.38)	73(79.35)	<0.001
Cumulative incidence GVHD % (95% CI)				
Grade II-IV aGVHD at 100 days	15.8(11.10-20.50)	13.2(7.52-18.88)	19.8(11.37-28.23)	0.192
Grade III-IV aGVHD at 100 days	5.0(2.06-7.94)	3.0(0.06-5.94)	8.1(2.22-13.98)	0.078
cGVHD at 2 years	9.8(5.49-14.11)	7.6(2.90-12.30)	12.1(4.65-19.55)	0.449
Death, n (%)	87(36.40)	56(38.10)	31(33.70)	0.492
Causes of death				
Relapse, n (%)	53 (22.17)	31(21.09)	22(23.91)	–
No remission, n (%)	3(1.26)	1(0.68)	2(2.17)	–
Graft failure, n (%)	2(0.84)	0(0.00)	2(2.17)	–
Infection, n (%)	24(10.04)	21(14.29)	3(3.26)	–
aGVHD, n (%)	1(0.42)	0(0.00)	1(1.09)	–
cGVHD, n (%)	3(1.26)	2(1.36)	1(1.09)	–
Pulmonary thrombi embolism, n (%)	1(0.42)	1(0.68)	0(0.00)	–
Relapse n (%)	63(26.36)	36(24.49)	27(29.35)	0.407
Overall survival % (95% CI)				0.232
At 1 year	70.6(64.72-76.48)	67.2(59.56-74.84)	76.1(67.48-84.72)	
At 2 years	64.7(58.43-70.97)	63.3(55.07-71.53)	68.2(58.60-77.80)	
At 3 years	60.9(54.24-67.56)	58.5(49.29-67.71)	65.1(55.10-75.10)	
Cumulative incidence of relapse % (95% CI)				0.760
At 1 year	20.5(14.81-26.18)	21.8(14.35-29.24)	18.5(9.88-27.12)	
At 2 years	27.4(20.94-33.87)	26.5(17.68-35.32)	28.3(18.30-38.29)	
At 3 years	28.1(21.47-34.80)	26.5(17.68-35.32)	29.8(19.80-39.80)	
Disease-free survival (95% CI)				0.356
At 1 year	66.5(58.46-74.54)	62.6(54.76-70.44)	72.8(63.78-81.82)	
At 2 years	58.0(51.53-64.47)	55.7(46.88-64.52)	61.9(51.90-71.90)	
At 3 years	57.3(50.64-63.96)	55.7(46.88-64.52)	60.4(50.20-70.59)	
GVHD/relapse free survival % (95% CI)				0.747
At 1 year	62.3(56.22-68.38)	60.5(52.66-68.34)	66.3(56.70-75.90)	
At 2 years	53.8(47.14-60.46)	53.4(44.58-62.22)	54.3(45.11-65.49)	
At 3 years	52.0(45.14-58.86)	51.5(42.29-60.71)	52.8(42.41-63.18)	
Cumulative incidence of treatment-related mortality% (95% CI)				0.102
At 1 year	13.0(8.46-17.48)	15.6(9.37-21.91)	8.7(2.81-14.57)	
At 2 years	14.6(9.51-19.70)	17.8(10.56-25.06)	9.8(3.31-16.25)	
At 3 years	14.6(9.51-19.70)	17.8(10.56-25.06)	9.8(3.31-16.25)	

aGVHD acute graft-versus-host disease, cGVHD chronic acute graft-versus-host disease, CMV cytomegalovirus, CI confidence interval.

### Infection and complications

One hundred and fifty patients (62.76%) developed CMV viremia after transplantation. The CIs of CMV viremia by day +180 and +360 were 64.2 ± 3.2% and 64.8 ± 3.2%, respectively. Compared with lower-dose ATG group, higher-dose ATG group significantly increased the incidence of CMV viremia (52.38% *vs* 79.35%, *P*<0.001, **Table 2**). Three patients developed CMV-associated diseases (2 with pneumonia and 1 with enteritis) in the lower-dose ATG group and five patients (3 with pneumonia and 2 with enteritis) in the higher-dose ATG group. One patient died of CMV-associated pneumonia in the higher-dose ATG group. Forty-six patients (19.25%) suffered from severe pneumonia (bacterial pneumonia in 40 patients, fungal pneumonia in 6 patients, shown in **Table 2**).

### Acute and chronic GVHD

**Figure 1:**
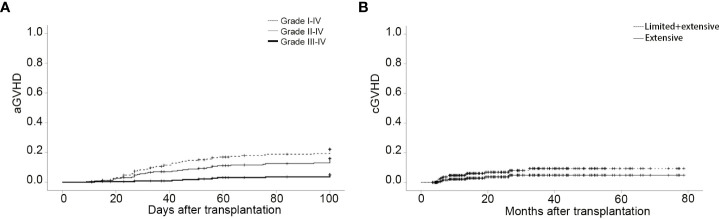
CIs of aGVHD and cGVHD. **(A)** The 100-day CI of grade I-IV aGVHD, II-IV aGVHD and III-IV aGVHD. **(B)**The CI of cGVHD and extensive cGVHD.

In this study, 231 patients were evaluated for aGVHD at 100 days. The total incidence of aGVHD was 21.21%. The incidences of grade I and II-IV aGVHD were 7.79% and 13.42%, respectively. The numbers of patients who occurred grade II aGVHD involvement of isolated skin, skin and gut, liver, and only gut were 14, 2, 1, and 3, respectively. Three patients developed grade III aGVHD and eight patients occurred grade IV aGVHD. The 100-day CIs of grade I-IV aGVHD, II-IV aGVHD, and III-IV aGVHD were 22.0 ± 2.8%, 15.8 ± 2.4%, and 5.0 ± 1.5%, respectively (**Figure 1A**). In this study, cGVHD was observed in 20 of 209 evaluable patients, including 12 patients with limited cGVHD and 8 patients with extensive cGVHD. The overall CIs of cGVHD at one year, two years, and three years were 7.7 ± 1.9%, 9.8 ± 2.2%, and 12.3 ± 2.7%, respectively. The CIs of extensive cGVHD at one year, two years, and three years were 2.6 ± 1.1%, 4.1 ± 1.5%, and 5.2 ± 1.9%, respectively (**Figure 1B**). There was no difference between PBSC and a mixture of BM and PBSC as graft in CI of grade II-IV aGVHD (15.5 ± 3.4%*vs* 16.2 ± 3.6%, *P*=0.878, **Supplementary Figure 2A**). Different conditioning regimens had no significant effect on CI of grade II-IV aGVHD (MAC:15.4 ± 4.7% *vs* IMC:16.0 ± 2.9%, *P*=0.958, **Supplementary Figure 2B**). Higher CI of grade II-IV aGVHD was observed in female to male than in others in donor/recipient gender match (26.2 ± 6.8% *vs* 13.4 ± 2.6%, *P*=0.035, **Supplementary Figure 2C**). Stem cell source, different conditioning regimens, and donor/recipient gender match didn’t show significantly statistical effect on cGVHD (**Supplementary Figures 2D–F**). Comparable CIs of grade II-IV aGVHD (ATG ≤ 6mg/kg: 13.2 ± 2.9%, ATG>6mg/kg:19.8 ± 4.3%, *P*=0.192) and cGVHD at two years (ATG ≤ 6mg/kg:7.6 ± 2.4%, ATG>6mg/kg:12.1 ± 3.8%, *P*= 0.449) were observed in different ATG groups (**Table 2**). Donor/recipient gender match (female to male) was the only risk factor for aGVHD in multivariate analysis. No risk factors were found in multivariate analysis of cGVHD (**Table 3**).

**Table 3:** Multivariate analysis for acute and chronic GVHD.

variable	HR	95%CI	*P* value
**Cumulative incidence of grade II-IV aGVHD**			
ATG dose (ATG>6mg/kg *vs* ATG ≤ 6mg/kg)	1.684	0.773-3.667	0.189
Donor/recipient gender match (female to male *vs* others)	2.134	1.015-4.485	0.046
Stem cell source (PBSC+BM *vs* PBSC)	0.910	0.423-1.959	0.810
Risk stratification (intermediate *vs* high)	1.234	0.494-3.084	0.652
Conditioning regimen (IMC *vs* MAC)	0.672	0.262-1.727	0.409
Recipient age	0.975	0.946-1.006	0.113
Disease type			
AML *vs* ALL	0.808	0.356-1.835	0.611
MDS *vs* ALL	1.136	0.348-3.705	0.833
**Cumulative incidence of cGVHD**			
ATG dose (ATG>6mg/kg *vs* ATG ≤ 6mg/kg	1.753	0.617-4.982	0.292
Donor/recipient gender match: female to male	1.039	0.333-3.240	0.948
Stem cell source (PBSC+BM *vs* PBSC)	0.530	0.191-1.470	0.222
Risk stratification (intermediate *vs* high)	4.561	0.586-35.524	0.147
Conditioning regimen (IMC *vs* MAC)	3.271	0.684-15.645	0.138
Recipient age	1.014	0.976-1.053	0.477
Disease type			
AML *vs* ALL	1.452	0.538-3.918	0.461
MDS *vs* ALL	1.028	0.206-5.141	0.973

aGVHD acute graft-versus-host disease, ALL acute lymphocytic leukemia, AML acute myeloid leukemia, ATG antithymocyte globulin, BM bone marrow, cGVHD chronic acute graft-versus-host disease, CI confidence interval, HR hazard ratio, IMC intensity myeloablative conditioning, MAC myeloablative conditioning, MDS myelodysplastic syndrome, PBSC peripheral blood stem cells.

### Relapse

**Figure 2:**
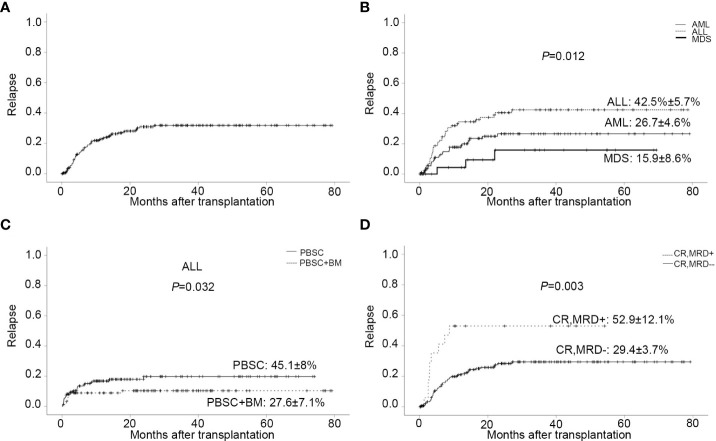
Relapse. **(A)** CI of relapse. **(B)** CI of relapse in patients with different disease types. **(C)** CI of relapse in ALL patients with different stem cell source. **(D)** CI of relapse in patients with MRD negative/positive before transplantation.

**Figure 3:**
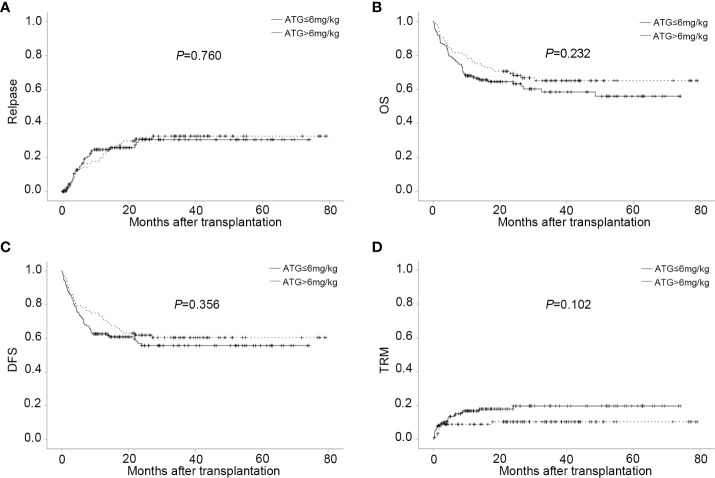
CIs of post-transplant outcome in different ATG doses groups. **(A)** CI of relapse in different ATG doses groups. **(B)** CI of OS in different ATG doses groups. **(C)** CI of DFS in different ATG doses groups. **(D)** CI of TRM in different ATG doses groups.

Sixty-three patients relapsed after transplantation, with a median time of 168 days (range,13 to 811). In relapsed patients, 26 AML, 34 ALL, and 3 MDS were included. In all leukemia-relapsed patients, all the other patients relapsed in the bone marrow, except for one patient who relapsed in the central nervous system. By the time of follow-up, 53 patients died of leukemia or tumor progression or chemotherapy-related complications and 10 patients were still alive, with a median survival time of 343 days (range,170 to 811). The overall CI of relapse was 20.5 ± 2.9% at one year, 27.4 ± 3.3% at two years, and 28.1 ± 3.4% at three years, respectively (**Figure 2A** and **Table 2**). The overall CI of relapse did not show statistical difference between patients in the intermediate-risk group and those in the high-risk group (34.0 ± 7.1% *vs* 31.0 ± 3.9%, *P*=0.766). In all patients, ALL patients had higher overall CI of relapse than AML and MDS patients (42.5% ± 5.7% *vs* 26.7 ± 4.6% *vs* 15.9 ± 8.6%, *P*=0.012, **Figure 2B**). For all patients, there was no statistical difference between PBSC and the combination of BM and PBSC as graft in relapse (36.8 ± 4.9% *vs* 25.9 ± 4.5%, *P*=0.103). However, in subgroup analysis, for ALL patients, result showed that the CI of relapse had significant difference in different graft sources (PBSC: 45.1 ± 8%, PBSC+BM: 27.6 ± 7.1%, *P*=0.032, **Figure 2C**). The results showed that patients in MRD positive had higher recurrence rate than patients in MRD negative (MRD+: 52.9 ± 12.1%, MRD-: 29.4 ± 3.7%, *P*=0.003, **Figure 2D**). The comparable probabilities occurred in CI of relapse between lower ATG(≤6mg/kg) and higher ATG(>6mg/kg) (*P*=0.760, **Figure 3A** and **Table 2**). In multivariate analysis, the combination of BM and PBSC as graft had a beneficial influence on relapse. Patients with MRD positive or diagnosed with ALL before transplantation had higher risk of relapse (**Supplementary Table 2**).

### OS, DFS, TRM, and GRFS

Eighty-seven patients died after transplantation with a median time of 139 days (range,3 to 1453). The main causes of death were relapse (22.17%) and infection (10.04%) shown in **Table 2**. The CI of OS for all patients at one year, two years, and three years were 70.6 ± 3.0%, 64.7 ± 3.2%, and 60.9 ± 3.4%, respectively (**Table 2**). The 3-year probability of OS was significantly lower for patients in transfusion of PBSC as graft than those in transfusion of BM combined with PBSC (54.0 ± 4.8% *vs* 69.3 ± 4.7%, *P*= 0.023). There was no statistically different between the intermediate-risk group and high-risk group in the 3-year of OS (64.7 ± 7.6% *vs* 59.8 ± 3.9%, *P*=0.256).

The probability of DFS for all patients at one year, two years, and three years were 66.5 ± 3.1%, 58.0 ± 3.3%, and 57.3 ± 3.4%%, respectively. Thirty-four patients died of TRM in this study with a median time of 51 days (range, 3 to 719). Pneumonia was the main reason of death. The overall CI of TRM was 13.0 ± 2.3% at one year, 14.6 ± 2.6% at two years, and 14.6 ± 2.6% at three years. The CI of GRFS was 62.3% ± 3.1% at one year, 53.8% ± 3.4% at two years, and 52.0% ± 3.5% at three years (**Table 2**). Regardless of lower-dose or higher-dose ATG group, no significant statistical difference occurred in OS, DFS, GRFS and TRM (**Figures 3B–D** and **Table 2**).

In multivariate analysis, patient had a poor prognosis on OS (HR=2.025,95% CI:1.050-3.904, *P*=0.035), DFS (HR=1.893, 95% CI:1.010-3.546, *P*=0.046) and TRM (HR=3.131, 95% CI:1.172-8.369, *P*=0.023) when age was over 50 years at transplantation. The combination of BM and PBSC as graft had a positive impact on OS (HR=0.575, 95% CI: 0.369-0.896, *P*=0.014) and DFS (HR=0.566, 95% CI: 0.374-0.855, *P*=0.007). Days of neutrophil engraftment (≥16 days) was associated with lower DFS (HR=0.566,95% CI: 1.141-3.409, *P*=0.015) and higher TRM (HR=2.217,95% CI: 0.989-4.968, *P*=0.053). Grade III-IV aGVHD was associated with lower OS (HR=2.488,95% CI:1.185-5.222, *P*=0.016) and higher TRM (HR=3.638, 95% CI: 1.368-9.671, *P*=0.010). The conditioning regimen IMC had an adverse impact on DFS (HR=1.861,95% CI: 1.111-3.119, *P*=0.018) and TRM (HR=3.721, 95% CI:1.282-10.800, *P*=0.016). No remission before transplantation was a common risk factor for OS, DFS, and TRM. Comparing with ALL patients, AML and MDS patients had better OS (AML *vs* ALL : HR=0.605, 95% CI:0.385-0.951, *P*=0.029; MDS *vs* ALL : HR=0.293,95% CI: 0.116-0.739, *P*=0.009) (**Supplementary Table 2**).

## Discussion

In the present retrospective study, basiliximab was administrated in addition to the ATG-based GVHD prophylaxis regimen in 239 patients who were diagnosed with intermediate- or high-risk hematologic malignancies and had received haplo-HSCT with a median follow-up of 2.4 years (range, 0.8-6.6). The efficacy of this similar protocol in high-risk hematologic malignancies has been explored (28–30). However, with respect to the experience of these groups, the higher number of patients, the longer follow-up of our study, the use of more intensive conditioning regimens, the different graft sources as well as the different dosages and types of ATG provide relevant novelty to the present report. Herein, this combined GVHD prophylaxis strategy could result in very favorable rates of acute and chronic GVHD and achieve satisfactory results of 3-year OS 60.9% with acceptable relapse rates and TRM. Remarkably, lower dose of ATG (≤6mg/kg) combined with basiliximab was associated with less CMV viremia but comparable GVHD preventive effect and survival rates.

Haploidentical donors provide the benefits of rapid and near universal donor availability. However, immunological barriers resulting from the high degree of HLA-mismatch in the haplo-HSCT settings were formidable (39, 40). *In vivo* T-cell depletion or modulation with ATG (the Beijing protocol) or PTCy (the Baltimore protocol) has been the basis for the development of multiple novel GVHD prophylaxis approaches used in haplo-HSCT. The clinical benefit of incorporating ATG or PTCy as GVHD prophylaxis in haplo-HSCT varied among studies, which could be related to the dose and formulation of ATG or PTCy administration, the conditioning regimen, the donor type, the stem cell source, the concomitant immunosuppressive medications and the background hematological malignancy (15, 41–50). We summarized several studies on the effects of ATG-based, PTCy-based, and two schemes combined GVHD prophylaxis (4–7, 12, 42, 43, 45, 47, 48, 51–54) (**Supplementary Table 3**). Comparable incidences of grade II-IV aGVHD (18%-42%) and severe cGVHD (10%-23%) were shown in ATG-based and PTCy-based prophylaxis. Meanwhile, PTCy-based regimen appears to be more effective in the incidence of grade III-IV aGVHD (5%-14%). Additionally, a novel regimen of combining ATG and PTCy after haplo-HSCT for hematological malignancies showed promising activity with grade II–IV aGVHD of 17%-26% and grade III–IV aGVHD of 3.2%-6.9%. It is worth noting that in our study, we observed that GVHD prophylaxis using the described ATG plus basiliximab combination results in very low rates of both acute and chronic GVHD, with estimated incidences of grade II-IV aGVHD, grade III-IV aGVHD and 3-year extensive cGVHD of 15.8%, 5% and 5.2%, respectively. The rates of clinically significant grade III–IV aGVHD and extensive cGVHD were significantly decreased, a benefit that has not been achieved in most of the studies using standard ATG regimen and PBSC. Accordingly, the effect of the use of ATG and basiliximab on the incidence of GVHD converted into the reduced risk of TRM and improved GRFS. Compared with PTCy-based studies, although our study showed lower severe aGVHD, lower TRM, and higher GRFS, this could be due to the younger baseline characteristics of the patients (47, 48, 51, 55). Older patients who receive reduced conditioning are often associated with more severe infections, GVHD, and recurrence, which results in lower GRFS. Therefore, clinical studies with larger sample sizes and older patients are needed to confirm the effectiveness.

Furthermore, our results observed that the female to male combination results in a higher incidence of severe acute GVHD than other donor-recipient sex combinations, which coincides with the results of others (5, 56, 57), but it did not translate into any higher TRM. Nevertheless, the incidence of GVHD was not influenced by the stem cell source, the types of hematological malignancy or intensity of conditioning regimens in multivariate analysis. Importantly, the incidence of GVHD in our study using unmanipulated PBSC combined or not with BM with intensified myeloablative or myeloablative conditioning was comparable to that in previous studies reported for similar GVHD prophylaxis, using unmanipulated BM haplo-HSCT with myeloablative or reduced intensity conditioning (29, 30). Thus, we concluded that the effect of this enhancing immunosuppression strategy may be independent of stem cell source and the intensity of conditioning regimen.

Infectious complications remain the major factors affecting overall survival and are central to advances seeking to improve haplo-HSCT. As far as we know, *in vivo* T-cell depletion with ATG results in delayed immune reconstitution, leaving patients vulnerable to severe infections, including viral reactivations with CMV or Epstein-Barr virus (EBV), bacterial infection, as well as infection-related deaths (13, 15, 58). The incidence of CMV viremia was 62.76% in our study, which was comparable with ATG-based regimens (60-78.6%) (5, 28, 30, 43, 59–61). Despite pre-emptive therapy of ganciclovir or foscarnet, 3% patients still progress to CMV disease. Antiviral drug resistance and treatment-limiting side effects remain major challenges. Letermovir was approved for prophylaxis of CMV-infection and -disease in adult CMV-seropositive recipients after allogeneic HSCT and demonstrated lower risk of CMV-infection than placebo without apparent safety concerns (62). Letermovir has a novel mechanism of action that inhibits CMV DNA synthesis at a late step by targeting the pUL56 subunit of the terminase enzyme complex (63–66). Because of a unique mechanism of action that is distinct from ganciclovir and other CMV DNA polymerase inhibitors, letermovir has the potential for treating multidrug-resistant CMV. We believe that with additional clinical efficacy data, this medication could emerge as a primary option for the prevention and treatment of CMV in the immunocompromised patient population.

As for the use of PTCy in haplo-HSCT for hematologic malignancies, a major concern is the high relapse rate, which is up to 50% (9, 11, 67–69). While the relapse rate at 1-year and 3-year were 20.5% and 28.1%, respectively, which was comparable to those in previous studies, ranging from 15% to 34.5% depending on the diagnosis and the disease risk (4, 5, 55, 70). In line with the published data, patients suffering from ALL showed a higher risk of relapse (51, 55). Different from ATG, basiliximab binds specifically to the IL-2R of activated T cells and only selectively eliminates the alloreactive donor lymphocytes, without affecting the resting T cells (24), which is important for preventing infectious complications. It was also reported that antibody mediated CD25 blockade may be useful to promote anti-tumor immunity (25). This may account for the comparable incidences of CMV viremia and relapse between our haplo-HSCT setting using ATG plus basiliximab and other regimens using ATG alone to prevent GVHD. Thus, we could conclude that ATG combined with basiliximab was complementary integration to reinforce GVHD prophylaxis without increasing infections and significantly compromising disease control.

The limitations associated with the use of ATG as a protocol for GVHD prophylaxis include the profound immune deficiency and higher risk of infectious complications, depending on the dose of ATG administered (15, 61, 71–73). To date, the optimal dose of ATG balancing the efficacy of GVHD prophylaxis and the risk of severe infections has not been established for haplo-HSCT. Hang et al. has documented that 6 mg/kg ATG applied in haplo-HSCT was related to a faster recovery of T cell reconstitution and lower incidence of EBV infection but a higher rate of severe GVHD and GVHD related death than 10 mg/kg ATG (15, 61, 74). Recently, a multicenter randomized study comparing two different doses of ATG (7.5 and 10 mg/kg) as GVHD prophylaxis for haplo-HSCT, showed that patients receiving 7.5 mg/kg ATG had a lower incidence of EBV and CMV DNAemia and a similar incidence of aGVHD and cGVHD compared with those receiving 10.0 mg/kg (75). In our haplo-HSCT system, we also made an initial attempt to investigate whether low doses of ATG, 6mg/kg and below, were more beneficial in the outcome of haplo-HSCT. Consistently with the mentioned results, the reduced incidence of CMV DNAemia was found in the lower dose arm(≤6mg/kg), compared to the higher dose arm (>6mg/kg). Surprisingly, there was no difference noted with regard to the occurrence of acute and chronic GVHD between the lower dose and higher dose groups. In addition, in the context of haplo-HSCT with similar GVHD prophylaxis, the incidence of GVHD in the lower dose arm seems to be better or equivalent to that in previous studies using higher dose of ATG, ranging from 6 to 9 mg/kg, or using different source of ATG (ATG-Fresenius, 20 mg/kg) (28–30). These findings suggest that lower dose ATG has sufficient efficacy for GVHD prophylaxis and minimizes the risk of infection when used in combination with basiliximab. Furthermore, long-term transplant outcomes did not show any significant differences between the two groups concerning disease recurrence, TRM, OS and DFS. It’s worth noting that a beneficial effect of TRM was not seen in the lower dose arm despite the lower incidences of CMV viremia and the comparable morbidity and mortality associated with GVHD. This may attribute to the effective preemptive treatment for CMV DNAemia at our center. Although survival benefit has not been achieved, it might bring a potential economic benefit as well as less side effects of preemptive therapies in the patients receiving lower dose ATG. In general, in our study, a lower dose ATG(≤6mg/kg) in combination with basiliximab was as effective as higher doses for GVHD prevention after haplo-HSCT. A higher dose did not confer any additional benefit; conversely it appeared to be associated with increased CMV DNAemia. Based on these results, a lower dose ATG(≤6mg/kg) seems to be more appropriate for balancing infection control and GVHD prophylaxis when used in combination with basiliximab. A prospective randomized trial would be required to reach a definite conclusion on the superior efficacy of lower dose ATG plus basiliximab regimen in haplo-HSCT.

Indeed, our study was limited by its retrospective nature and the heterogeneity of the sample size. To some extent, the dose of ATG was related to HLA mismatch and the impact of ATG dose on the incidence of GVHD may be confounded by HLA mismatch. Although several studies have shown that HLA mismatch has little effect on GVHD in haplo-HSCT, this issue cannot be ignored (5, 28, 76, 77). Thus, further prospective and randomized control studies are needed. In addition, more detailed analyses are needed to get information on other virus infections including EBV and immune reconstitution in this haplo-HSCT circumstance. However, we included a relatively large patient number with a rather long follow-up, which allowed us to reliably estimate the impact of ATG in conjunction with basiliximab on long-term clinical outcomes at our center.

In conclusion, the evidence reported in this paper indicates that the combination of ATG with basiliximab is a feasible and effective protocol to promote protection against GVHD and improve haploidentical transplant outcomes in the context of both intensified myeloablative and myeloablative conditioning regimens. Moreover, the combination with a lower dose of ATG has shown to be safer and equally effective than the original one with a higher dose, in preventing GVHD. We conclude that the optimal scheme of using a combined regimen of ATG and basiliximab is that administration of lower dose ATG(≤6mg/kg), which could exert a synergistic activity to reduce the risk of GVHD without increasing severe infection, particularly CMV viremia.

## Data availability statement

The original contributions presented in the study are included in the article/**Supplementary Material**. Further inquiries can be directed to the corresponding authors.

## Ethics statement

The studies involving human participants were reviewed and approved by the Ethical Committee of Huazhong University of Science and Technology. Written informed consent to participate in this study was provided by the participants’ legal guardian/next of kin.

## Author contributions

LX and WS designed the study and enrolled patients. HY and ZH analyzed the data and wrote the paper. ZH and YT were responsible for the data collection. All authors contributed to the article and approved the submitted version.

## Funding

This work was supported by the National Natural Science Foundation of China (No.81974003, No. 81500168, No. 81974007), the National Key R&D Program of China (Grant No 2019YFC1316204), and Collaborative Innovation Center of Hematology of China.

## Acknowledgments

We are very grateful to all the participants at the institute who provided information in this study.

## Conflict of interest

The authors declare that the research was conducted in the absence of any commercial or financial relationships that could be construed as a potential conflict of interest.

## Publisher’s note

All claims expressed in this article are solely those of the authors and do not necessarily represent those of their affiliated organizations, or those of the publisher, the editors and the reviewers. Any product that may be evaluated in this article, or claim that may be made by its manufacturer, is not guaranteed or endorsed by the publisher.

## Abbreviations

aGVHD, acute GVHD; ALL, acute lymphoblastic leukemia; AML, acute myeloid leukemia; ATG, antithymocyte globulin; BM, bone marrow; cGVHD, chronic GVHD; CI, cumulative incidence; CIs, cumulative incidences; CI, confidence interval; CsA, cyclosporin A; CMV cytomegalovirus; CR, complete remission; DSA, donor-specific antibodies; DFS, disease-free survival; EBV epstein-Barr virus; GRFS, GVHD/relapse-free survival; GVHD, graft versus host disease; HLA, human leukocyte antigen; HR, hazard ratio; Haplo-HSCT, haploidentical hematopoietic stem cell transplant; IMC, intensity myeloablative conditioning; MAC, myeloablative conditioning; MDS, myelodysplastic syndrome; MMF, mycofenolate mofetil; MRD, minimal residual disease; MRD+, minimal residual disease positive; MRD-, minimal residual disease negative; MTX, methotrexate; NR, no remission; OS, overall survival; PBSC, peripheral blood stem cells; PTCy, post-transplant cyclophosphamide; TRM, transplant-related mortality.
