# Supplementary material for: Lower dose of ATG combined with basiliximab for haploidentical hematopoietic stem cell transplantation is associated with effective control of GVHD and less CMV viremia
Source: Front Immunol. 2022 Nov 15;13:1017850. doi: 10.3389/fimmu.2022.1017850 (PMC9705727; doi:10.3389/fimmu.2022.1017850)
Supplement: Supplementary file 1 [file DataSheet_1.docx]

**Supplementary material file**

**Figure 1. GVHD prophylaxis diagram.**

**Figure 2. CIs of II-IV aGVHD and cGVHD.**

(A.D) CIs of grade II-IV aGVHD and cGVHD for stem cell source. (B.E) CIs of grade II-IV aGVHD and cGVHD for conditioning regimens. (C.F) CIs of grade II-IV aGVHD and cGVHD for donor/recipient gender match.

**Table 1. The exact doses of ATG in 239 patients**

Abbreviations: ATG antithymocyte globulin.

**Table 2. Multivariate analysis for relapse, OS, DFS, and TRM**

Abbreviations: aGVHD acute graft-versus-host disease, ALL acute lymphocytic leukemia, AML acute myeloid leukemia, BM bone marrow, CI confidence interval, CR complete remission, DFS disease-free survival, HR hazard ratio, IMC intensity myeloablative conditioning, MAC myeloablative conditioning, MDS myelodysplastic syndrome, MRD+ minimal residual disease positive, MRD- minimal residual disease negative, NR no remission, OS overall survival, PBSC peripheral blood stem cells, TRM transplant-related mortality.

**Table 3. Comparison of the incidence of GVHD in ATG-based, PTCy-based, and combined GVHD prophylaxis**

Abbreviations: ATG antithymocyte globulin, CsA cyclosporin A, GVHD graft-versus-host disease, MMF, mycofenolate mofetil, MTX methotrexate, PTCy post-transplant cyclophosphamide, Ref reference, Siro Sirolimus, Tacro tacrolimus.

**Figure 1.**


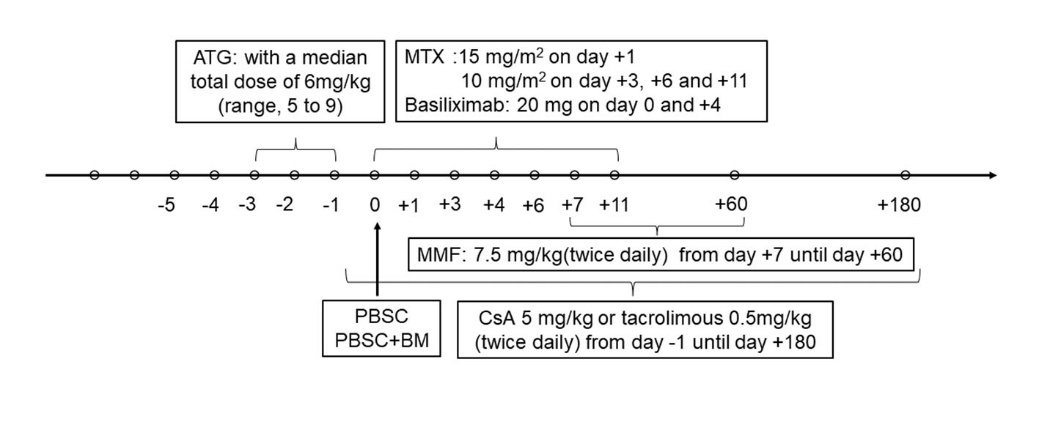


**Figure 2.**


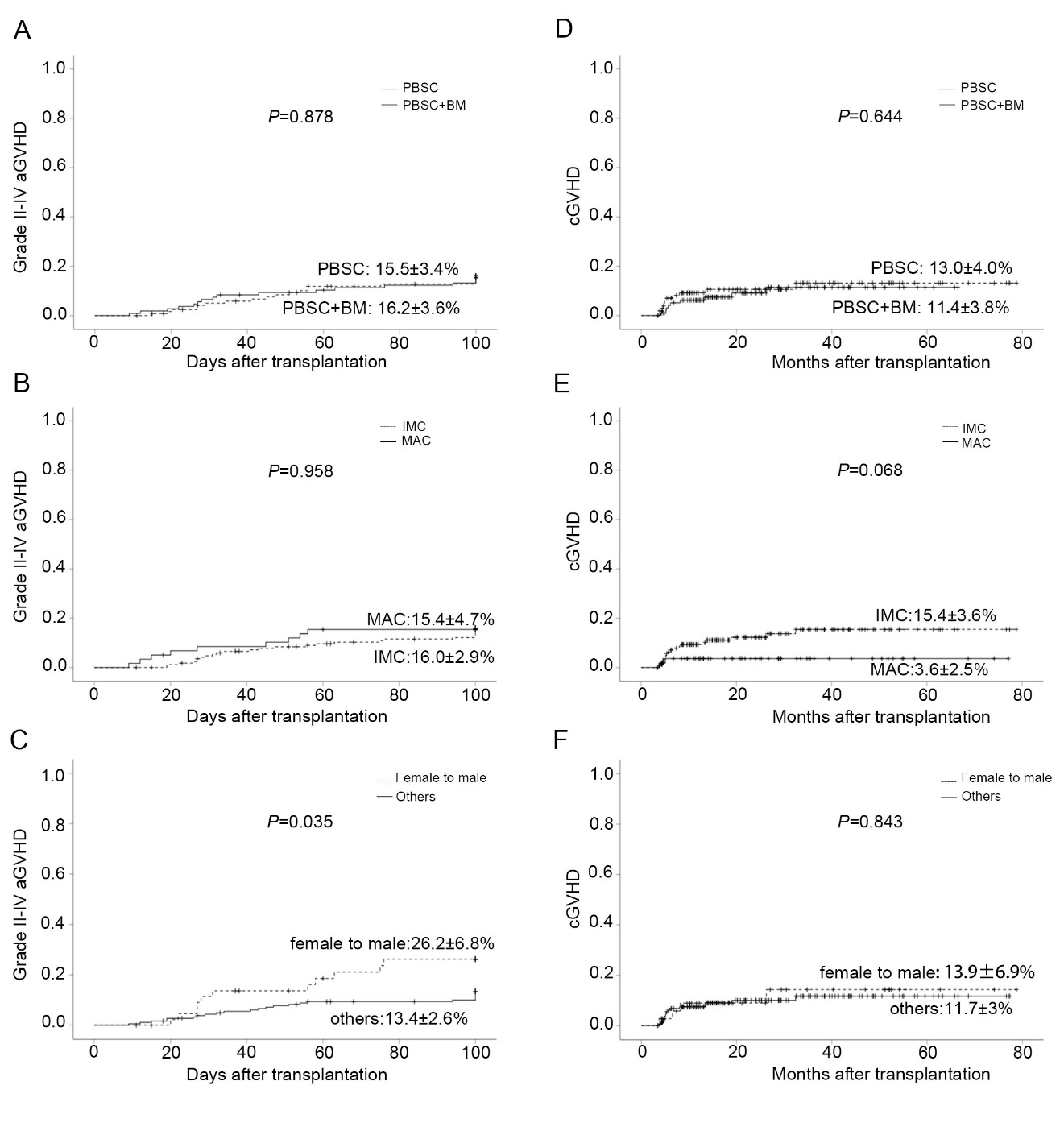


Table 1. The exact doses of ATG in 239 patients

| Groups | total ATG dose | cases | administration time |
| --- | --- | --- | --- |
| ATG≤6mg/kg | 5mg/kg | 7 | from day -2 to day -1 |
|  | 6mg/kg | 140 | from day -3 to day -1 |
| ATG >6mg/kg | 7.5 mg/kg | 53 | from day -3 to day -1 |
|  | 9 mg/kg | 39 | from day -3 to day -1 |

Table 2. Multivariate analysis for relapse, OS, DFS, and TRM.

| Variables | HR | 95%CI | *P* value |
| --- | --- | --- | --- |
| **Relapse** |  | | |
| Disease type |  | | |
| AML vs ALL | 0.547 | 0.327-0.916 | 0.022 |
| MDS vs ALL | 0.241 | 0.070-0.828 | 0.024 |
| Disease status before transplantation  CR, MRD+ vs CR, MRD - | 2.870 | 1.383-5.954 | 0.005 |
| Stem cell source (PBSC+BM vs PBSC) | 0.577 | 0.344-0.969 | 0.038 |
| **OS** |  |  |  |
| Disease status before transplantation |  |  |  |
| CR, MRD+ vs CR, MRD- | 1.957 | 0.993-3.855 | 0.052 |
| NR vs CR, MRD- | 2.569 | 1.353-4.880 | 0.004 |
| Disease type |  |  |  |
| AML vs ALL | 0.605 | 0.385-0.951 | 0.029 |
| MDS vs ALL | 0.293 | 0.116-0.739 | 0.009 |
| Grade III-IV aGVHD | 2.488 | 1.185-5.222 | 0.016 |
| Stem cell source (PBSC+BM vs PBSC) | 0.575 | 0.369-0.896 | 0.014 |
| Age (≥50y vs <50y) | 2.025 | 1.050-3.904 | 0.035 |
| **DFS** |  |  |  |
| Disease status before transplantation |  | | |
| MRD+ vs MRD- | 2.021 | (1.030-3.968) | 0.041 |
| NR vs MRD- | 1.829 | (1.043-3.209) | 0.035 |
| Days of myeloid engraftment (≥16 days vs <16 days) | 1.972 | (1.141-3.409) | 0.015 |
| Stem cell source (PBSC+BM vs PBSC) | 0.566 | (0.374-0.855) | 0.007 |
| Conditioning regimen (IMC vs MAC) | 1.861 | (1.111-3.119) | 0.018 |
| Age (≥50y vs <50y) | 1.893 | (1.010-3.546) | 0.046 |
| **TRM** |  | | |
| Disease status before transplantation |  |  |  |
| MRD+ vs MRD- | 0.495 | (0.067-3.679) | 0.492 |
| NR vs MRD- | 2.873 | (1.318-6.263) | 0.008 |
| Conditioning regimen (IMC vs MAC) | 3.721 | (1.282-10.800) | 0.016 |
| Age (≥50y vs <50y) | 3.131 | (1.172-8.369) | 0.023 |
| Days of myeloid engraftment (≥16 days vs <16 days) | 2.217 | (0.989-4.968) | 0.053 |
| Grade III-IV aGVHD | 3.638 | (1.368-9.671) | 0.010 |

Table 3. Comparison of the incidence of GVHD in ATG-based, PTCy-based, and combined GVHD prophylaxis

| GVHD prophylaxis | Ref | cases | GVHD prophylaxis | Grade II-IV aGVHD  at 100 days | Grade III-IV aGVHD  at 100 days | cGVHD | extensive cGVHD |
| --- | --- | --- | --- | --- | --- | --- | --- |
| ATG-based GVHD prophylaxis | 1 | 231 | ATG+CsA+MMF+MTX | 36% | 10% | 42% at 1 year | 12% at 1 year |
|  | 2 | 742 | ATG+CsA+MMF+MTX | 43% | 14% | 53% at 2 years | 23% at 2 years |
|  | 3 | 130 | ATG+CsA+MMF+MTX | 33% | 15% | 37% at 3 years | 17% at 3 years |
|  | 4 | 115 | ATG+CsA/Tacro/Siro±MTX±MMF± basiliximab | 21% | 13% | 28% at 2 years | 13% at 2 years |
|  | 5 | 125 | ATG+CsA+MMF+MTX | 36% | 18% | 44% at 2 years | 16% at 2 years |
|  | 6 | 196 | ATG+calcineurin inhibitor or Siro +MMF | 34% | 14% | 32% at 2 years | 13% at 2 years |
|  | 7 | 176 | ATG+CsA+MMF+MTX | 27% | 8% | 42% at 3 years | 10% at 3 years |
| PTCy-based GVHD prophylaxis | 4 | 193 | PTCY+CsA/Tacro+MMF | 31% | 5% | 34% at 2 years | 9% at 2 years |
|  | 6 | 313 | PTCY+calcineurin inhibitor+ MMF | 31% | 12% | 27% at 2 years | 11% at 2 years |
|  | 7 | 44 | PTCY+CsA+MMF+MTX | 18% | 7% | 26% at 3 years | 6% at 3 years |
|  | 8 | 32 | PTCY+ Siro+MMF | 19% | 9% | 47% at 1 year | 19% at 1 year |
|  | 9 | 451 | PTCY+calcineurin inhibitor+MMF | 21-38% | 4-14% | 32-36% | 10-12% |
|  | 10 | 687 | PTCY+Tacro/CsA+MMF | 25%-42% at 6 months | - | 20%-41% at 2 years | - |
|  | 11 | 148 | PTCY+CsA +MMF | 18% | 4% | 20% | - |
| Combination of ATG and PTCy | 5 | 114 | PTCY+CsA+MMF+ATG+MTX | 26% | 5% | 30% at 2 years | 17% at 2 years |
|  | 12 | 50 | PTCY+CsA +ATG | 20% | 5%, | 10% | - |
|  | 13 | 32 | PTCY+CsA+MMF+ATG | 19% | 7% | 19% at 6 months | - |
|  | 14 | 47 | PTCY+ CsA +ATG | 17% | 6% | 17% | 15% at 1 year |

References:

1. Wang Y, Liu QF, Xu LP, Liu KY, Zhang XH, Ma X, et al. Haploidentical vs identical-sibling transplant for AML in remission: a multicenter, prospective study. Blood (2015) 125(25):3956-3962. doi: 10.1182/blood-2015-02-627786
2. Wang Y, Liu D-H, Liu K-Y, Xu L-P, Zhang X-H, Han W, et al. Long-term follow-up of haploidentical hematopoietic stem cell transplantation without in vitro T cell depletion for the treatment of leukemia: nine years of experience at a single center. Cancer (2013) 119(5):978-985. doi: 10.1002/cncr.27761
3. Huang WR, Li HH, Gao CJ, Bo J, Li F, Dou LP, et al. Haploidentical, unmanipulated G-CSF-primed peripheral blood stem cell transplantation for high-risk hematologic malignancies: an update. Bone Marrow Transplant (2016) 51(11):1464-1469. doi: 10.1038/bmt.2016.166
4. Ruggeri A, Sun Y, Labopin M, Bacigalupo A, Lorentino F, Arcese W, et al. Post-transplant cyclophosphamide versus anti-thymocyte globulin as graft-versus-host disease prophylaxis in haploidentical transplant. Haematologica (2017) 102(2):401-410. doi: 10.3324/haematol.2016.151779
5. Wang Y, Wu DP, Liu QF, Xu LP, Liu KY, Zhang XH, et al. Low-dose post-transplant cyclophosphamide and anti-thymocyte globulin as an effective strategy for GVHD prevention in haploidentical patients. J Hematol Oncol (2019) 12(1):88. doi: 10.1186/s13045-019-0781-y
6. Lorentino F, Labopin M, Fleischhauer K, Ciceri F, Mueller CR, Ruggeri A, et al. The impact of HLA matching on outcomes of unmanipulated haploidentical HSCT is modulated by GVHD prophylaxis. Blood Advances (2017) 1(11):669-680. doi: 10.1182/bloodadvances.2017006429
7. Tang F, Xu Y, Chen H, Xu L, Zhang X, Wang Y, et al. Comparison of the clinical outcomes of hematologic malignancies after myeloablative haploidentical transplantation with G-CSF/ATG and posttransplant cyclophosphamide: results from the Chinese Bone Marrow Transplantation Registry Group (CBMTRG). Science China. Life sciences (2020) 63(4):571-581. doi: 10.1007/s11427-019-9594-7
8. Bejanyan N, Pidala JA, Wang X, Thapa R, Nishihori T, Elmariah H, et al. A phase 2 trial of GVHD prophylaxis with PTCy, sirolimus, and MMF after peripheral blood haploidentical transplantation. Blood Advances (2021) 5(5):1154-1163. doi: 10.1182/bloodadvances.2020003779
9. Ruggeri A, Labopin M, Bacigalupo A, Gülbas Z, Koc Y, Blaise D, et al. Bone marrow versus mobilized peripheral blood stem cells in haploidentical transplants using posttransplantation cyclophosphamide. Cancer (2018) 124(7):1428-1437. doi: 10.1002/cncr.31228
10. Bashey A, Zhang MJ, McCurdy SR, St Martin A, Argall T, Anasetti C, et al. Mobilized Peripheral Blood Stem Cells Versus Unstimulated Bone Marrow As a Graft Source for T-Cell-Replete Haploidentical Donor Transplantation Using Post-Transplant Cyclophosphamide. Journal of clinical oncology: official journal of the American Society of Clinical Oncology (2017) 35(26):3002-3009. doi: 10.1200/JCO.2017.72.8428
11. Bacigalupo A, Dominietto A, Ghiso A, Di Grazia C, Lamparelli T, Gualandi F, et al.. Unmanipulated haploidentical bone marrow transplantation and post-transplant cyclophosphamide for hematologic malignanices following a myeloablative conditioning: an update. Bone Marrow Transplant (2015) 50 Suppl 2:S37-S39. doi: 10.1038/bmt.2015.93
12. Law AD, Salas MQ, Lam W, Michelis FV, Thyagu S, Kim DDH, et al. Reduced-Intensity Conditioning and Dual T Lymphocyte Suppression with Antithymocyte Globulin and Post-Transplant Cyclophosphamide as Graft-versus-Host Disease Prophylaxis in Haploidentical Hematopoietic Stem Cell Transplants for Hematological Malignancies. Biology of blood and marrow transplantation: journal of the American Society for Blood and Marrow Transplantation (2018) 24(11):2259-2264. doi: 10.1016/j.bbmt.2018.07.008
13. Yang J, Jiang J, Cai Y, Li S, Wan L, Zhu J, et al. Low-dose anti-thymocyte globulin plus low-dose posttransplant cyclophosphamide as graft-versus-host disease prophylaxis in haploidentical peripheral blood stem cell transplantation combined with unrelated cord blood for patients with hematologic malignancies: a prospective, phase II study. Bone Marrow Transplant (2019) 54(7):1049-1057. doi: 10.1038/s41409-018-0382-3
14. Salas MQ, Law AD, Lam W, Al-Shaibani Z, Loach D, Kim DDH, et al. Safety and Efficacy of Haploidentical Peripheral Blood Stem Cell Transplantation for Myeloid Malignancies Using Post-transplantation Cyclophosphamide and Anti-thymocyte Globulin as Graft--Host Disease Prophylaxis. Clin Hematol Int (2019) 1(2):105-113. doi: 10.2991/chi.d.190316.003
